# The consequences of replacing wildlife with livestock in Africa

**DOI:** 10.1038/s41598-017-17348-4

**Published:** 2017-12-08

**Authors:** Gareth P. Hempson, Sally Archibald, William J. Bond

**Affiliations:** 1 Centre for African Ecology, School of Animal, Plant and Environmental Sciences, University of the Witwatersrand, Johannesburg, 2050 South Africa; 2 South African Environmental Observation Network (SAEON), Pretoria, 0001 South Africa

## Abstract

The extirpation of native wildlife species and widespread establishment of livestock farming has dramatically distorted large mammal herbivore communities across the globe. Ecological theory suggests that these shifts in the form and the intensity of herbivory have had substantial impacts on a range of ecosystem processes, but for most ecosystems it is impossible to quantify these changes accurately. We address these challenges using species-level biomass data from sub-Saharan Africa for both present day and reconstructed historical herbivore communities. Our analyses reveal pronounced herbivore biomass losses in wetter areas and substantial biomass increases and functional type turnover in arid regions. Fire prevalence is likely to have been altered over vast areas where grazer biomass has transitioned to above or below the threshold at which grass fuel reduction can suppress fire. Overall, shifts in the functional composition of herbivore communities promote an expansion of woody cover. Total herbivore methane emissions have more than doubled, but lateral nutrient diffusion capacity is below 5% of past levels. The release of fundamental ecological constraints on herbivore communities in arid regions appears to pose greater threats to ecosystem function than do biomass losses in mesic regions, where fire remains the major consumer.

## Introduction

Large mammal herbivore populations have been radically altered across the globe since the late Pleistocene^1^. Recent research has highlighted the staggering extent of herbivore extinctions, particularly of the largest-bodied species, and how human pressures have devastated populations of surviving species^2–5^. This loss of native species has wide-ranging ecological consequences^6,7^, and has produced a surge of interest in how rewilding initiatives can restore herbivores and their cascading effects on ecosystems^8–10^. However, livestock densities in many parts of the world often approach or exceed herbivore densities before the Pleistocene extinctions^11,12^. This raises the important question of whether livestock restore ecological processes, by serving as functionally comparable replacements for extirpated species, or if their influence converts landscapes into novel ecosystems^13^.

Africa was least affected by the Pleistocene extinctions^14^, and has the largest remaining area of untransformed (uncultivated) land^15^. However, livestock have been progressively introduced into herbivore communities over thousands of years, with pastoralism practiced throughout the continent from at least 2,000 years ago^16,17^. Over the last few centuries colonial hunters have decimated indigenous herbivore and predator communities^18^, with the simultaneous increase in human population, and improved disease and vector control measures (e.g. tsetse fly suppression), meaning that livestock now vastly outnumber wildlife^19,20^. Today, most African herbivore communities have a much narrower range of body sizes and diets than in the past, which in turn reduces the range in the amount, quality, and type of plant material that can be consumed in particular ecosystems^21^. As a consequence, the spatial distribution of herbivore biomass across the continent will also have been altered^22^.

Recent studies have emphasised how large mammal herbivores can strongly modify ecosystems through effects on fire prevalence^23–25^, woody cover^26–28^ and biogeochemical cycling^29–31^. However, all of these processes are subject to environmental control, and with herbivore communities also being structured by environmental gradients^22,32^, responses will inevitably be contingent on factors like rainfall and soils^19,20,33,34^. A full appreciation of these impacts, and their use as drivers of rewilding programs and climate mitigation policies, requires that we quantify them. Fire management policies, sustainable woodland harvesting rates, and greenhouse gas accounting efforts all require that we have some understanding of how these processes operated in the past ecosystems which we have so dramatically transformed^35–37^. Africa is arguably the continent best suited to exploring these effects, because although it is no less ‘distorted’ than other ecosystems globally, there is a rich knowledge about the ecology of both wildlife and livestock on the continent across a range of environments (this is one of the few places where quantifiable data on the impacts of near-intact megafaunal assemblages are possible). Moreover, this information has been used to reconstruct and map past herbivore densities on the continent^12^, providing an opportunity to quantify some of the ecological impacts of these trophic disruptions.

Here we explore how the form and the intensity of herbivory pressure has changed across Africa by contrasting livestock-dominated present day herbivore communities with reconstructed past herbivore communities. The past biomass surface comprises pre-colonial era (i.e. about 1,000 years ago) species-level biomass estimates for 92 extant native herbivores^12^. Cattle, goats and sheep were included in the present day livestock biomass estimates^38^, with remnant wildlife populations approximated by filtering past biomass using information on a region’s conservation status, human footprint index^39^, and extent of area converted to cropland^20^. Species-level information on body size, diet, gut type and water dependence^12^ were used to gauge how shifts in herbivore community composition would influence ecological processes such as fire, tree-grass dynamics, carbon emissions and nutrient distribution. Broad patterns of global relevance emerge from our analysis of how herbivore community turnover is shaped by environmental conditions. The results provide a more nuanced model for understanding these changes across other continents, and represent essential validation data for global vegetation models which aim to reproduce the ecological impacts of herbivores^40^.

## Results and Discussion

### Herbivore biomass change

**Figure 1:**
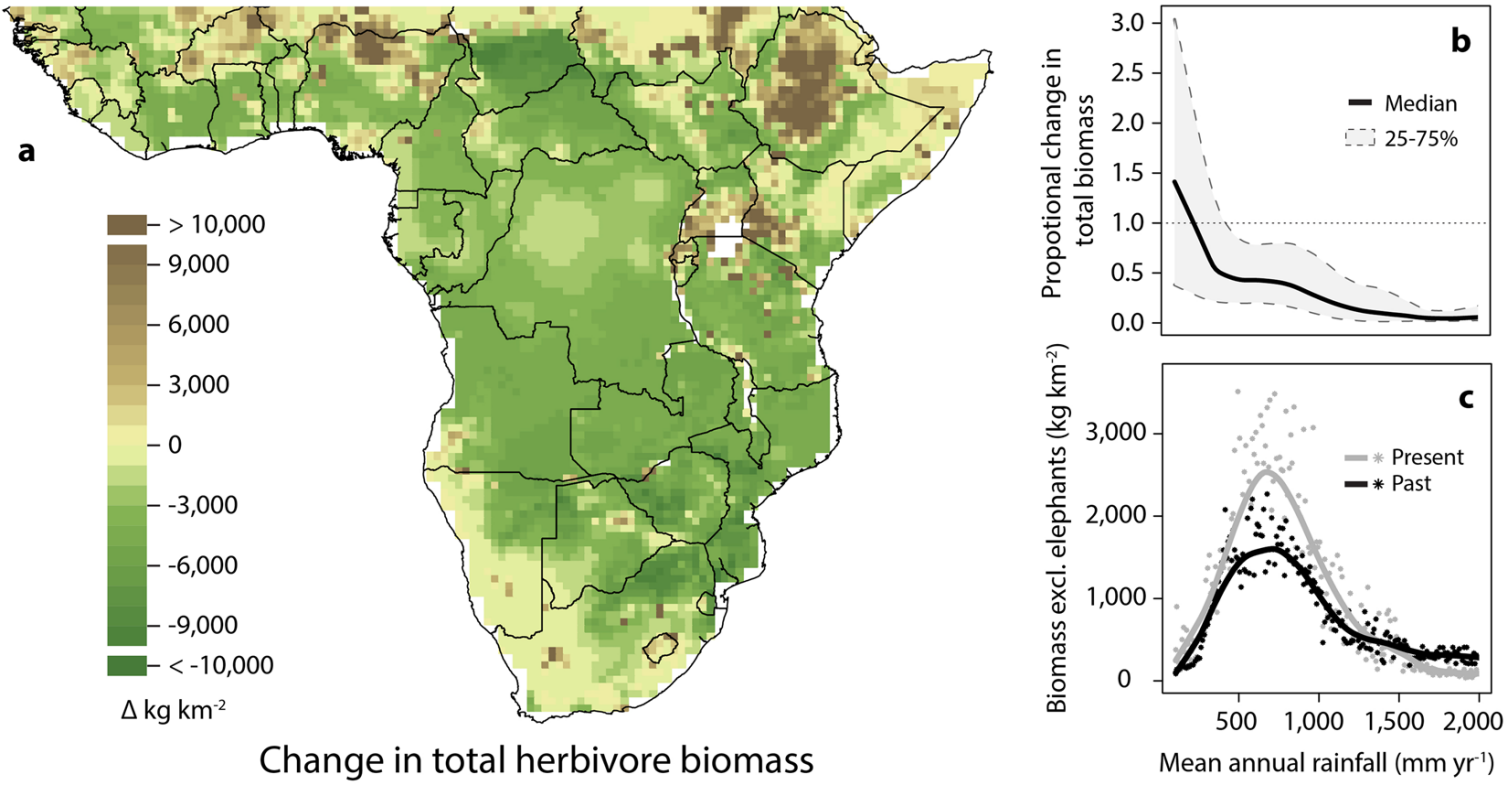
Patterns of herbivore biomass change in Africa. (**a**) Absolute change in herbivore biomass (present – past), and (**b**) proportional change (present/past) in herbivore biomass in relation to mean annual rainfall. (**c**) Herbivore biomass excluding elephants in relation to mean annual rainfall. Points (**c**) represent median values for 10 mm yr^−1^ rainfall intervals and are shown with locally weighted scatterplot smoothing regression lines (**b**,**c**). Figure 1(c) is similar to Fig. 1 in Hempson *et al*.^12^, but differs by present biomass including both livestock and wildlife, and by being quantified at 0.5° spatial resolution. The map was generated using R version 3.3.3^93^ (www.R-project.org) and QGIS 2.4.0.^94^ (www.qgis.org).

Total herbivore biomass has decreased across most of Africa (Fig. 1a), with maximum declines exceeding 10,000 kg km^−2^. The only regions where herbivore biomass has increased are arid – below 500 mm yr^−1^ – with average changes only being positive below 250 mm yr^−1^ (Fig. 1b). These arid regions are also the least predictable, with either large increases or declines observed in many ecosystems. This is attributable to low historical biomass in these arid regions, but is also because humans can support artificially high herbivore abundance in dry areas by providing supplementary water and forage^11^. The average current biomass of herbivores falls below 10% of historical levels in the wettest parts of Africa ( > 1,300 mm yr^−1^). Elephants once dominated herbivore biomass in these regions^12^, and their extirpation by hunting is largely responsible for these overall declines. Excluding elephants from the analysis (Fig. 1c) reveals that livestock match or exceed past wildlife biomass in areas with rainfall up to about 1,500 mm yr^−1^, but cannot compensate for wildlife biomass in wetter regions.

### Herbivore functional turnover

**Figure 2:**
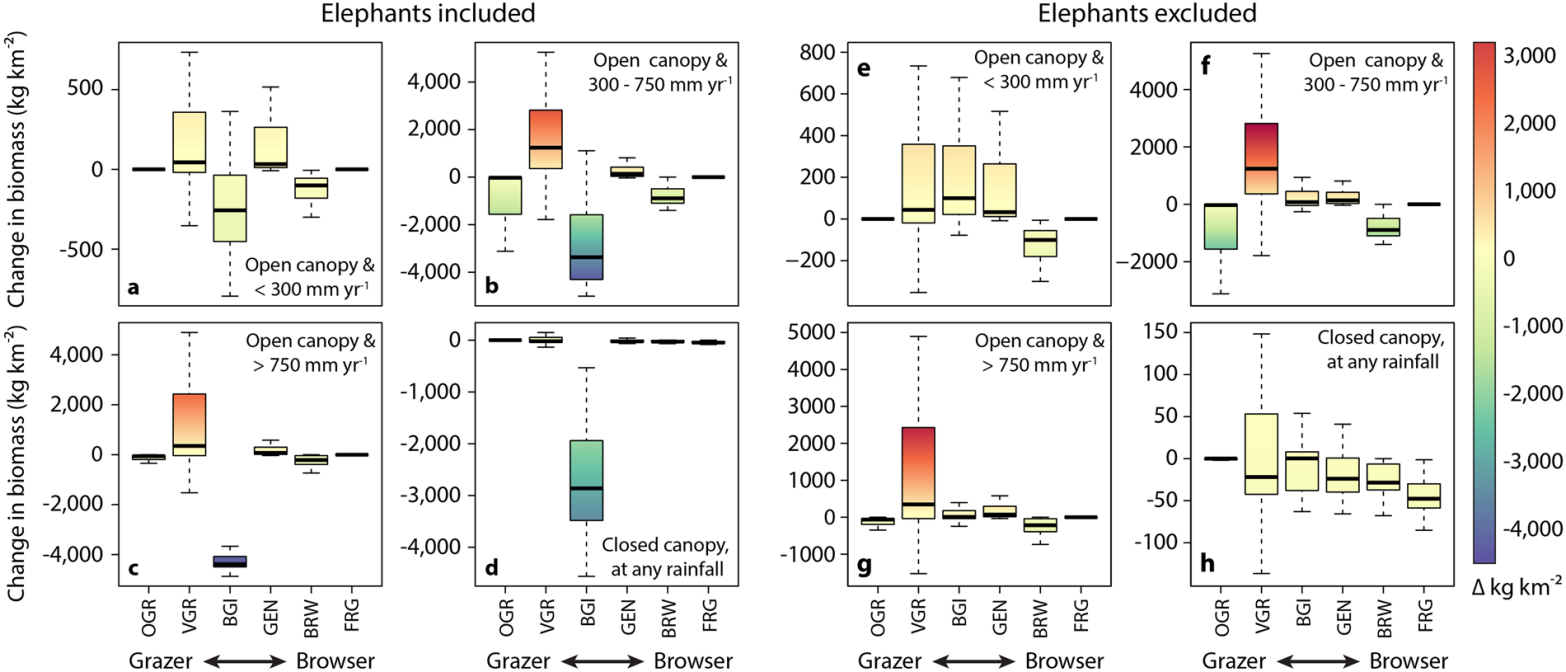
Herbivore biomass change by diet type. Biomass change is shown for open vegetation types for three mean annual rainfall categories: (**a**,**e**) < 300 mm yr^−1^, (**b**,**f**) 300–750 mm yr^−1^ and (**c**,**g**) > 750 mm yr^−1^. All closed canopy vegetation types are shown in (**d**,**h**). Elephants are browser-grazer intermediates and historically dominated herbivore biomass across the continent; changes in biomass are shown both including (**a**–**d**) and excluding (**e**–**h**) elephants. Livestock diet classifications: cattle = variable grazers, goats = generalists and sheep = browser-grazer intermediates^41^. Boxes show the median and interquartile range, and whiskers extend to the most extreme data point that is no further than the interquartile range. Median values are significantly different from zero (p = 0.05) in all cases. Dietary classifications follow Gagnon & Chew^41^: OGR = obligate grazer ( > 90% monocots, not variable), VGR = variable grazer (60–90% monocots, variable), BGI = browser-grazer intermediate (30–70% dicots and monocots, < 20% fruits), GEN = generalist ( > 20% of monocots, dicots and fruits), BRW = browser ( > 70% dicots) and FRG = frugivore ( > 70% fruits, little or no monocots).

Three livestock species dominate African rangelands, accounting for over 90% of current day herbivore biomass. The vast majority of the continent has thus experienced a substantial contraction of herbivore trait diversity. Fortuitously, cattle (water-dependent grazers), and goats and sheep (both medium-sized social mixed diets), are not novel herbivore functional types in African ecosystems^12^. Nonetheless, the relative abundance of e.g. different herbivore diet types (Fig. 2) has shown dramatic and varied shifts across rainfall and vegetation type categories (Supplementary Figure S1). Cattle, which are variable grazers (i.e. consuming 60–90% grass^41^), dominate herbivore biomass in regions with open vegetation types and rainfall < 300 mm yr^−1^ (Fig. 2b,c,f,g). The greatest relative contribution of sheep and goats to increased herbivore biomass is in the driest parts of the continent ( < 300 mm yr^−1^), reflecting their lower water dependence and more varied diets (Fig. 2a; goat effect is evident in Fig. 2e which excludes elephants). Closed canopy vegetation types have lost herbivore biomass across all dietary categories (Fig. 2d,h), although the decline in elephant populations makes the effect on browser-grazer intermediates particularly pronounced. Elephants appear to have strong effects on the density and diversity of saplings in African forests^42^, and also act as important seed dispersers^43^, and their widespread loss may thus have broad implications for forest community dynamics. The lack of an elephant analogue – and specialist browser and frugivore diet types – among livestock species also appears to hold important consequences for open-canopied African vegetation communities, for example by potentiating bush encroachment^33,44,45^ or reducing seed dispersal^46–48^.

### Fire-grazer competition

**Figure 3:**
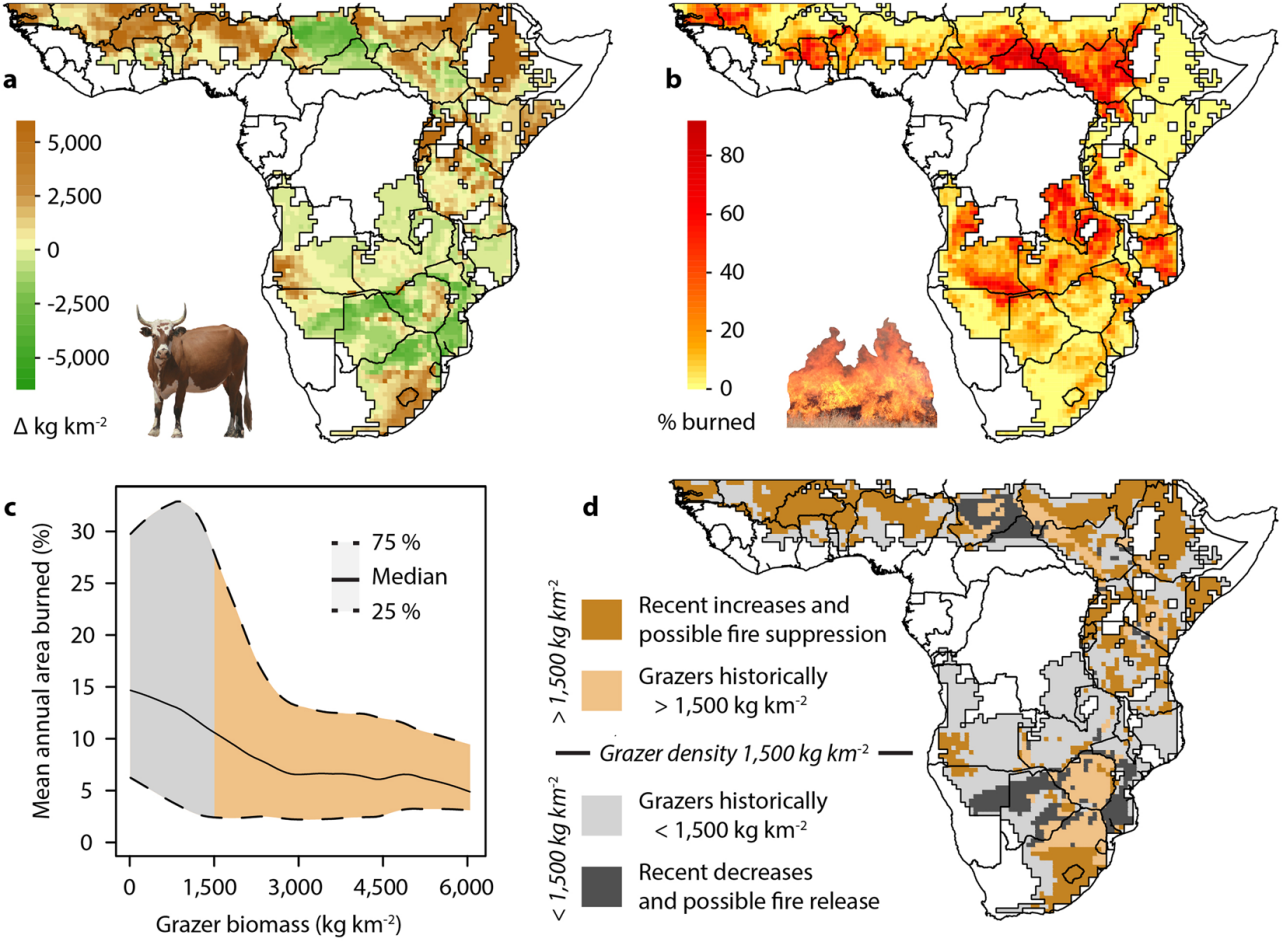
Fire prevalence in relation to grazer biomass. (**a**) Grazer biomass change (kg km^−2^), (**b**) mean annual burned area (%) and (**c**) the relationship between present day grazer biomass and mean annual burned area for savannas with annual rainfall between 300–1,300 mm yr^−1^. Panel (**d**) indicates whether grazer biomass has crossed a threshold value of 1,500 kg km^−2^ (corresponding to the colour bands in (**c**), above which fire-grazer competition has greater potential to reduce mean burned area^20^ (analysis restricted to areas with <40% woody cover). Regions with enhanced present day fire-grazer competition (i.e. dark brown areas in **d**) correspond closely with patterns of mean annual burned area (i.e. the match with lightly shaded areas in **b**), particularly in East and West Africa. Panel (**c**) was derived by fitting locally weighted scatterplot smoothing regression lines to median grazer biomass values at 20 kg km^−2^ intervals. The maps were generated using R version 3.3.3^93^ (www.R-project.org) and QGIS 2.4.0^94^ (www.qgis.org).

Conceptually, grazers can suppress fires when they consume so much grassy material in the wet season that there is insufficient fuel to carry fires in the dry season. The effect of grazers is therefore dependent both on the number of grazers and the productivity of the grass layer (i.e. more and/or larger-bodied grazers are needed in wetter areas to suppress fire^49^. At continental scales grazers have clear negative effects on fire prevalence in savannas above a density of 1,500 kg km^−2^ ^20^ (Fig. 3c). This suggests that current fire patterns are substantially influenced by changes in grazer communities (Fig. 3a) – both positively and negatively (Fig. 3d). The close match between regions exceeding this threshold (Fig. 3d) and patterns of annual burned area (Fig. 3b) suggests that fire has been strongly suppressed across vast expanses of Africa that previously would have had too few grazers to suppress grassy fires (dark brown regions in Fig. 3d). This large-scale evidence for fire-grazer interactions supports paleoecological data showing that losing large mammal grazers could lead to higher fire prevalence^23,24^. An important caveat, however, is that environmental conditions are important in shaping large mammal effects on ecosystems: grazers are predicted to have the greatest potential to alter grassland structure in a manner that excludes fire (e.g. by creating grazing lawns) where rainfall lies between 400–850 mm yr^−1^ ^50^. Consistent with this is the lack of evidence for change in fire prevalence in the mesic Miombo regions (>1,000 mm yr^−1^) spanning Angola to northern Mozambique (Fig. 3b,d). The most pronounced effects of fire release on other continents are thus likely to have occurred where fire has replaced herbivores as the dominant consumer in xeric savannas^20,51^, although the rainfall threshold at which this occurs may vary^52^.

### Woody cover

**Figure 4:**
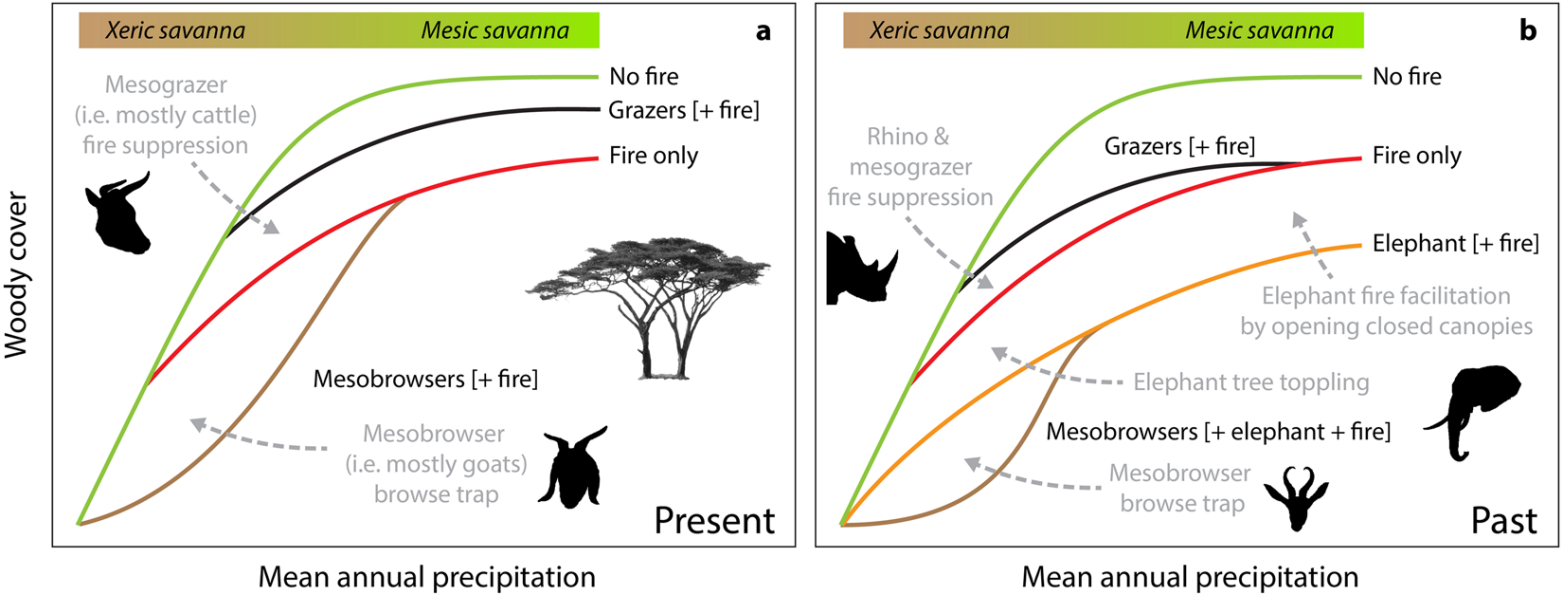
Conceptual model of the effects of herbivore community distortion on woody cover. Shifts in the biomass and functional composition of herbivore communities are likely to mean that their net effect on woody cover is different now (**a**) to in the past (**b**). The green line represents the maximum tree cover along a rainfall gradient in the absence of fire or herbivory following Sankaran *et al*.^95^. The red line represents the putative effect of fire on tree cover in the absence of herbivory. Grazers (black line) suppress fire by reducing fuel loads thereby increasing woody cover, while elephants (orange line) and mesobrowsers (brown line) have direct consumptive effects that reduce woody cover while also enhancing tree vulnerability to fire. Xeric to mesic savanna transitions are broadly aligned with the 650 mm yr^−1^ threshold revealed by Sankaran *et al*.^95^. Grey arrows associate particular herbivore influences with relevant parts of the rainfall-woody cover conceptual space. Two of the herbivore icons make a reappearance after previously featuring in Hempson *et al*.^12^.

Herbivores can have direct effects on woody cover, but can also act indirectly by altering local fire regimes^53,54^. Rainfall, and to a lesser extent soil nutrient status, mediates the influence of different herbivore functional groups via productivity-linked effects on woody plant growth rates^55^ and the quantity and quality of grass biomass^56^. We predict that the general pattern of herbivore community distortion should enhance woody cover in African savannas (Fig. 4), over and above the influence of elevated CO_2_-levels that are a global driver of woody encroachment^57–59^. The substantial contraction of elephant distribution ranges and population sizes is a well-recognised direct release on woody cover that has occurred widely across the continent^33,45,53^. This loss equates to reduced tree toppling^26^, but also includes indirect effects on fire by facilitating grass and thus fire spread into closed canopy vegetation types^60^, and an increase in the susceptibility of damaged adult trees to fire^61,62^. Moreover, the widespread increase in grazer densities (Fig. 3a) has likely reduced fire across much of the continent (Fig. 3d), resulting in a lower severity fire-trap that otherwise can limit woody recruitment^63^ (Fig. 4). Grazers may also promote woody recruitment by reducing grass competition^53^. However, there are also conditions where herbivore community distortion might have decreased woody cover: small-stock farming across the continent has increased the biomass of mesobrowsers (Fig. 2), which may suppress woody cover via an enhanced browse-trap effect^53,63^. The loss of white rhino from ecosystems and the associated increase in fire in the general landscape may also constrain tree cover in mesic savannas^49^, and suggests that losing megaherbivores might not always result in higher tree cover^7,27,28^.

### Greenhouse gas emissions

**Figure 5:**
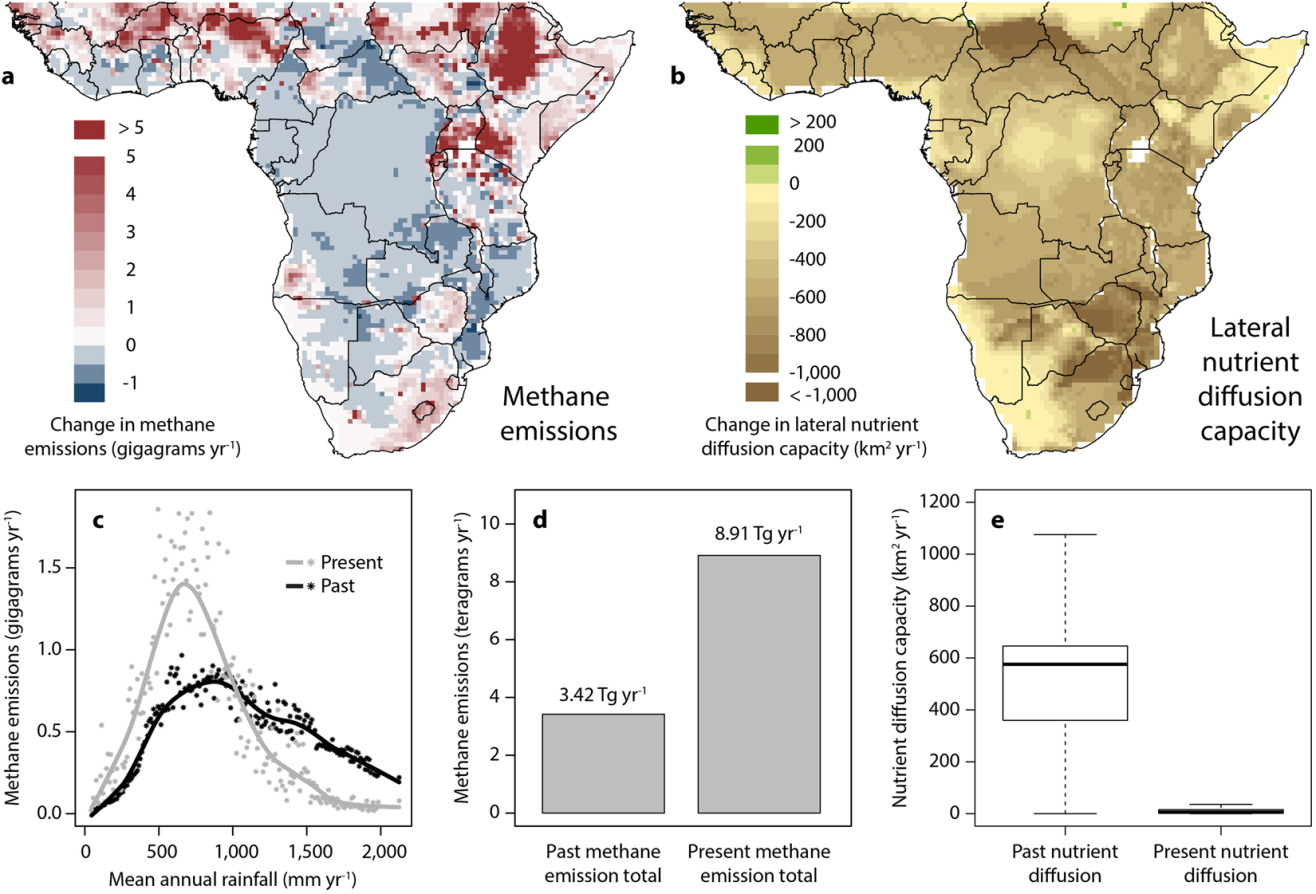
Herbivore effects on biogeochemical cycling. Shifts in herbivore community composition have altered (**a**) methane emissions and (**b**) lateral nutrient diffusion capacity across sub-Saharan Africa (both at 0.5° spatial grain). The relationship between rainfall and methane emissions (**c**) broadly reflects the overall dependence of herbivore biomass on rainfall (Fig. 1), with cattle-dominated communities now producing a higher peak in drier regions than in the past, but falling below past levels in wetter areas that were historically dominated by elephants. Total methane emissions for sub-Saharan Africa (**d**) are now considerably higher than in the past, while there have been marked decreases in nutrient diffusion capacity (**e**). In (**e**), box shows the median and interquartile range of nutrient diffusion capacity for all sub-Saharan Africa 0.5° cells, and whiskers extend to the most extreme data point which is no more than 1.5 times the interquartile range. The maps were generated using R version 3.3.3^93^ (www.R-project.org) and QGIS 2.4.0.^94^ (www.qgis.org).

The vegetation changes above will alter the carbon cycle and atmospheric CO_2_ levels through altering both above and below-ground biomass. Soil organic carbon has been shown to be remarkably resilient to grazing in tropical grassy ecosystems^64^ – only dropping off at extremely high grazing intensity. On the other hand, woody thickening due to replacement of browsers with grazers can increase above-ground carbon stocks. Neither of these impacts are well quantified spatially. It is easier to quantify the methane impacts of herbivore distortion, and as methane has 28 times the warming potential of CO_2_^65^, these are arguably more significant. The transition to livestock-dominated African herbivore communities has led to a net increase in methane emissions by enteric fermentation (Fig. 5). Herbivore methane emissions are strongly contingent on gut type (i.e. ruminant vs. non-ruminant) and body size: ruminants produce considerably more methane per unit body mass than non-ruminants, while larger animals produce more methane per unit body mass^37^. These functional trait details are important for understanding how a ruminant, cattle-dominated community – often with lower total biomass – can exceed methane emissions of a non-ruminant, elephant-dominated historical biomass in regions receiving rainfall below 1,000 mm yr^−1^ (Fig. 5c). Our analyses suggest that although approximately 50% of the sub-Saharan Africa land surface has experienced a decrease in methane emission levels (Fig. 5a), this is outweighed by substantial increases across large parts of the Sahel, Ethiopia and East Africa that combined produce a net increase from 3.4 to 8.9 Tg yr^−1^ (Fig. 5d). This estimate is broadly in line with recently revised estimates of methane emissions from livestock^66^ for the entire African continent (9.9 Tg), and constitutes approximately 15% of current global methane emissions (67.2 Tg). These calculations build on exciting recent attempts to quantify shifts in herbivore contributions to global carbon cycling^31^, but there remains much scope for improving these estimates with validation from detailed local studies. Our analysis differs from previous assessments^20,31,37^ by using considerably more nuanced herbivore biomass data, and by downscaling the contribution of elephants by using a body mass estimate that better reflects typical herd age structure (i.e. from ~4000 kg to 1725 kg^67^). Overall, our calculations suggest that Smith *et al*.’s^31^ global estimates of end-Pleistocene methane emission levels are probably too high, but highlight the clear need for further research to better understand herbivore contributions of this important non-CO_2_ greenhouse gas^68^.

### Lateral nutrient diffusion capacity

Recent theoretical studies have provided intriguing insights into how animals can disperse nutrients across ecosystems^29,30,36^. Importantly, animals can transport nutrients against passive movement gradients (e.g. upslope) and away from nutrient hotspots, thereby increasing overall landscape-level fertility patterns^69^. We extended recent allometric equation based approaches^30,36^ to quantify changes in nutrient diffusion capacity in Africa, but substituted the population density term in the models with our own independently-derived herbivore abundance surfaces to produce spatially-explicit estimates across the continent. Furthermore, we modified our present day nutrient diffusion estimates by scaling the daily movement range term for all species by an index of human influence^39^, with a further 50% reduction in day movement range imposed on livestock species. These modifications attempt to account for land use transformation effects on habitat connectivity, and also the effects of fences and night corralling on livestock movement (see Supplementary Figure S2 for estimates without day range constraints). The resulting surface confirms the widespread loss of the nutrient dispersing ecosystem service provided by large mammal herbivores in Africa^29,30,36^ (Fig. 5b), but projects a far more dramatic decline than in these previous estimates. Our results suggest that nutrient diffusion levels for sub-Saharan Africa are < 5% of past levels (Fig. 5e), and thus contrast strongly with Doughty *et al*.’s^36^ global assessment that suggests Africa retains 46% of its Pleistocene nutrient diffusion capacity. This is made all the more striking because previous assessments did not include contributions from livestock (further enhancing discrepancies between the studies), and suggests that while allometric estimates of herbivore population density may provide a useful first approximation of their influences, future projections should aim to incorporate information on how environmental conditions shape population densities.

### Ecosystem susceptibility

Mean annual rainfall plays a clear role in determining the form and extent of herbivore community distortion that occurs in African ecosystems. Mesic regions are most prone to overall biomass losses, while many arid regions have experienced considerable increases in herbivore biomass. Which end of this spectrum is of most concern? The associated turnover in herbivore functional type composition experienced in arid regions, largely in response to human interventions through water and supplemental feed provisioning and predator and disease control^11^, suggests that many of these regions may now be entering a novel state following the release of fundamental ecological constraints. Associated with the shifts in herbivore biomass and composition, populations are now also likely to be more sedentary, posing a further perturbation to vegetation dynamics that are generally more seasonally pulsed^70^. For example, year-round trampling and grazing may increase grass tuft mortality^71,72^, leading to increased soil erosion by wind and water, and reduced water infiltration due to soil compaction and greater run-off^73^. A global meta-analysis of the indirect effects of large mammal herbivores on ecosystems suggest these to be greatest in low productivity regions, despite the lower herbivore biomass in these regions^74^. This is explained by large mammal herbivores having the greatest potential to modify vegetation and hence habitats and food resources for other animal species in these regions^75^. Herbivore community distortion in arid regions may thus have many consequences beyond those of fire suppression and woody encroachment that we have explored here.

Mesic regions may be more buffered against herbivore losses due to fire having been the major consumer in these ecosystems over evolutionary timescales^20,51,76^. Despite general decreases in grazer biomass, historically low grazer abundance in these regions suggests that their impact on fire is likely to always have been minimal, meaning that fire prevalence is probably largely unaffected. The extirpation of elephants holds more substantial ecological implications, notably through enhanced woody thickening, and reduced lateral nutrient diffusion capacity in these typically highly leached, nutrient poor ecosystems^77^. On the other hand – humans are replacing some of the functions of elephants in these mesic systems through fuelwood harvesting^78^, and currently woody biomass is declining over much of the region in Africa^35^ – and in some instances to a state potentially more similar to what it was before elephant hunting^79^. Human hunting pressure is likely to remain the greatest threat to large mammal herbivores in Africa’s tropical forest ecosystems^80^, exacerbating current effects on seed dispersal, recruitment and vegetation structure in the forest understory^42,46,81^.

The general shift from migratory to resident herbivore populations, due to fences and land use change that act as barriers to movement, constitutes a ubiquitous but poorly quantified distortion of large mammal herbivore ecology. Only a handful of ecosystems now retain the diversity of functional seasonal resources necessary to support large, migratory herbivore populations that can exhibit the adaptive movement responses needed to persist at high densities in inherently variable ecosystems^82,83^. Populations that cannot migrate are more susceptible to droughts^71,72^ – periods during which intense defoliation of vegetation occurs – and will have lower mean population sizes with higher interannual variation^83^. These effects are most pronounced in climatically variable arid regions, but also highlight the considerable natural fluctuation in herbivore densities that occurs at local scales^84^, due to both movement and the intrinsic and extrinsic factors that regulate population size^32,85^.

### Implications

Currently there are several global initiatives afoot for carbon mitigation through reforestation (e.g. REDD + ^86^), altered fire regimes^87^, and livestock methane management^37,88^. The spatial data presented here for Africa give a slightly different perspective on the ecological appropriateness of some of these schemes – implying not only in the past there was extensive wood harvesting by elephant across large areas that are currently considered ‘deforested’, but that fire has been suppressed by livestock in a significant area of the continent. Moreover, rewilding schemes are likely to have consequences for national methane budgets, as large parts of Africa currently have methane ‘credits’ from the rampant destruction of their megaherbivores in the last few centuries^1,18^. Current African landscapes represent a continuum from pure livestock to pure wildlife. While livestock have been, and should continue to be an intrinsic part of African ecology, our data identify which processes and which environments are degraded by this shift in herbivore functional attributes, which should enable better interventions to restore these essential functions. Multispecies wildlife-livestock production systems are one such consideration that have long been advocated as a means to reinforce ecological resilience^19^ and support the unique biodiversity of the disturbance-maintained ecosystems that account for over half of Africa’s land surface^51,89,90^.

### Final remarks

Africa is effectively the world’s last laboratory for testing the effects of native large mammal herbivores on ecosystems. Even so, herbivore communities across Africa are massively transformed, with livestock now dominating the continent’s large mammal biomass. This raises an important question going forward as to where, globally, livestock serve to restore ecological processes, and where their influence converts landscapes into novel ecosystems^13^. Our knowledge about the spatial distributions of past herbivore communities is scarce, but although our results presented here for Africa remain necessarily coarse, they clearly demonstrate that using spatially-invariant mean values can result in under-, or over-estimation of how herbivore extinctions have altered important ecological processes. Merging insights from Africa may thus help to considerably refine projections of herbivore influences across other continents. The mounting evidence that large mammals matter should heighten support for Dirzo *et al*.’s^5^ call to elevate ‘defaunation’ to a status commensurate with that of ‘deforestation’ in wider society.

## Methods

### Herbivore biomass and environmental variables

Past biomass estimates were recalculated at quarter degree grid square resolution (0.5° × 0.5°) following the methods used in Hempson *et al*.^12^. Present day biomass estimates were updated from Archibald & Hempson^20^, and include both livestock^38^ and remnant wildlife population estimates. Livestock population estimates in Archibald & Hempson^20^ were updated with finer grained FAO data (0.0083° vs. 0.05°), which report higher livestock numbers for sub-Saharan Africa that better reflect total estimates for the region. Additional details are provided in the Supplementary Information. Change in herbivore biomass was calculated by subtracting past biomass values from present day biomass values, and was assessed for a range of different species and functional type groupings: total (Fig. 1a,b), total excluding elephants (Fig. 1c), diet type (following Gagnon & Chew^41^, and shown for four vegetation canopy cover-rainfall categories [Supplementary Figure S1]; with elephants included [Fig. 2a-d] and excluded [Fig. S2e-h]) and for grazer species (i.e. the combined biomass of obligate and variable grazers [Fig. 3]). Biomass change values are shown in relation to mean annual rainfall (WorldClim database, www.worldclim.org; accessed June 2013; Figs 1b,c and 5c). Mean annual area burned (Fig. 3b,c) was derived from the Global Fire Emissions Database 4.1 (GFED) 3.1 following van der Werf *et al*.^91^. Vegetation canopy cover classifications used in Fig. 2 were based on White^92^, with evergreen forests (mapping units: 1a, 2, 3, 4, 6, 8, 9), forest-grassland mosaics (50% of area of mapping units: 11a, 12, 17, 19a, 19b, 20, 65, 66) and mangrove forests (mapping unit 77) scored as closed canopy vegetation types and the remainder as open canopy systems. One-sample t-tests or else non-parametric sign tests were used to test whether the mean or median change in biomass was different from zero for each diet type in each rainfall × vegetation type category in Fig. 2.

### Methane emissions and lateral nutrient diffusion capacity

Methane emissions (kg indvidual^−1^ yr^−1^) were estimated separately for ruminant and non-ruminant species using body mass-methane output relationships from Smith *et al*.^37^: ruminant methane emissions = 10^−0.619 + 0.812 x log10(*BM*)^1.171^, and non-ruminant methane emissions = 10^−4.564 + 3.278 x log10(*BM*)^0.592^, where *BM* = body mass in kilograms. Lateral nutrient diffusion capacity (km^2^ yr^−1^) was calculated at 0.5° spatial grain based on the revised equation in Doughty *et al*.^36^, which is reformulated from the allometric relationships and the random walk-process outlined in Wolf *et al*.^30^ and Doughty *et al*.^29^ i.e. nutrient diffusion capacity = MR*PD*(DD*PR)^2^/(2*PR), where *MR* = metabolic rate (0.021*BM ^0.716^), *PD* = population density, *DD* = daily movement range (0.453*BM ^0.368^) and *PR* = food passage time (0.29*BM ^0.26^). We substituted our independently derived estimates of population density into the model instead of using the allometric relationship for *PD*, and used a body mass estimate of 1725 kg for elephants^67^. Daily movement range was rescaled (*DD*_r_) for present day nutrient diffusion capacity estimates using the human influence index^39^ (*HII*) i.e. *DD*_r_ = DD*(1–*HII*/72), where 72 is the maximum possible *HII* value. Livestock daily movement ranges (*DD*_rl_) were further reduced by 50% to account for the effects of fences and night corralling i.e. *DD*_rl_ = DD*(1–*HII*/72)*0.5. The datasets generated during and/or analysed during the current study are available from the corresponding author on reasonable request.

## Electronic supplementary material

Supplementary material
