# Supplementary material for: The consequences of replacing wildlife with livestock in Africa
Source: Sci Rep. 2017 Dec 8;7:17196. doi: 10.1038/s41598-017-17348-4 (PMC5722938; doi:10.1038/s41598-017-17348-4)
Supplement: Supplementary file 1 — Supplementary material [file 41598_2017_17348_MOESM1_ESM.pdf]

Supplementary Information

Hempson, Archibald & Bond – Scientific Reports – The consequences of replacing wildlife with livestock in Africa

Brief overview of methods used for calculating herbivore biomass surfaces. Historical herbivore biomass estimates were recalculated at quarter degree grid square resolution (0.5° x 0.5°) following the methods used by Hempson et al.[12]. Briefly, herbivore census data were obtained for protected areas across the continent, and filtered for quality. These data were used to build species-level statistical models of population density (individuals km^{-2}) in response to rainfall, soil nutrient status and vegetation type. These models were then used to estimate a species-level population density surface spanning their distribution range, with estimates being reduced in low preference (10%) and unsuitable (0%) habitat types within this range. Population density surfaces were converted to biomass surfaces (kg km^{-2}) using mean adult body mass. The derivation of the current herbivore biomass surfaces is detailed in Archibald & Hempson[20]. These surfaces include a livestock component (cattle, sheep and goats) and also remnant wildlife populations. Livestock data were obtained from the FAO[38] and aggregated to quarter degree grid square resolution. Remnant wildlife populations were estimated by filtering the historical herbivore biomass surface according to five levels of landscape change. These landscape change levels were assigned using protected area status, the human footprint index[39], and area converted to cropland. Elephant, black rhino and white rhino were only retained in the most intact landscapes, with their densities scaled to match country-level population estimates. All other species were subject to a size-biased filtering approach that reduced densities as landscape change increased, with larger species being more sensitive to change. Populations were eliminated if their downscaled density fell below 0.1 individuals km^{-2}.

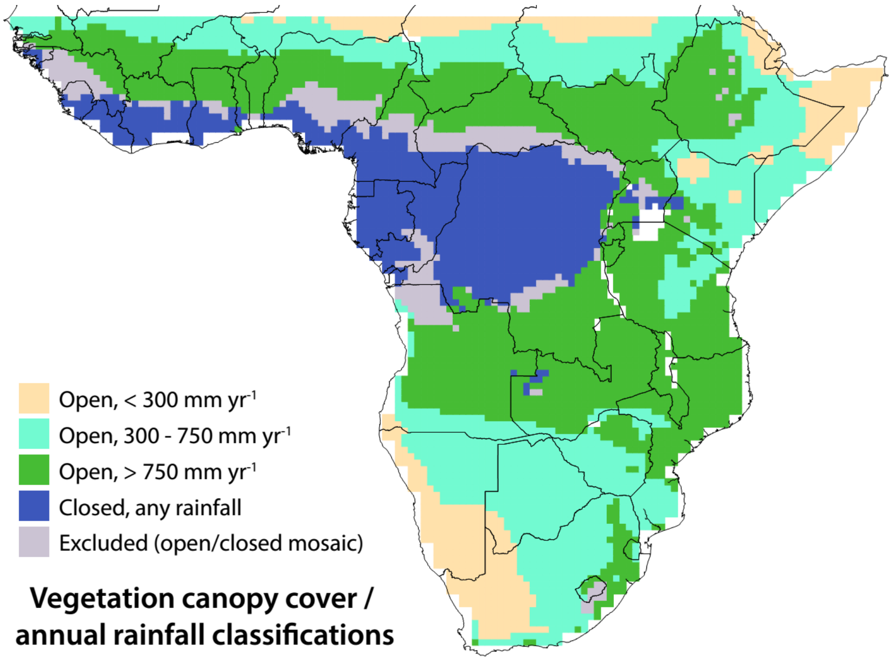

Figure S1. Vegetation canopy-rainfall classifications. Sub-Saharan Africa was characterized based on whether open or closed canopy vegetation types dominated a 0.5° x 0.5° grid square following White [91]. Regions dominated by open vegetation types were then further classified by rainfall based on whether they received < 300 mm yr^{-1}, 300–750 mm yr^{-1} or > 750 mm yr^{-1}. The map was generated using QGIS 2.4.0[42] (www.qgis.org).

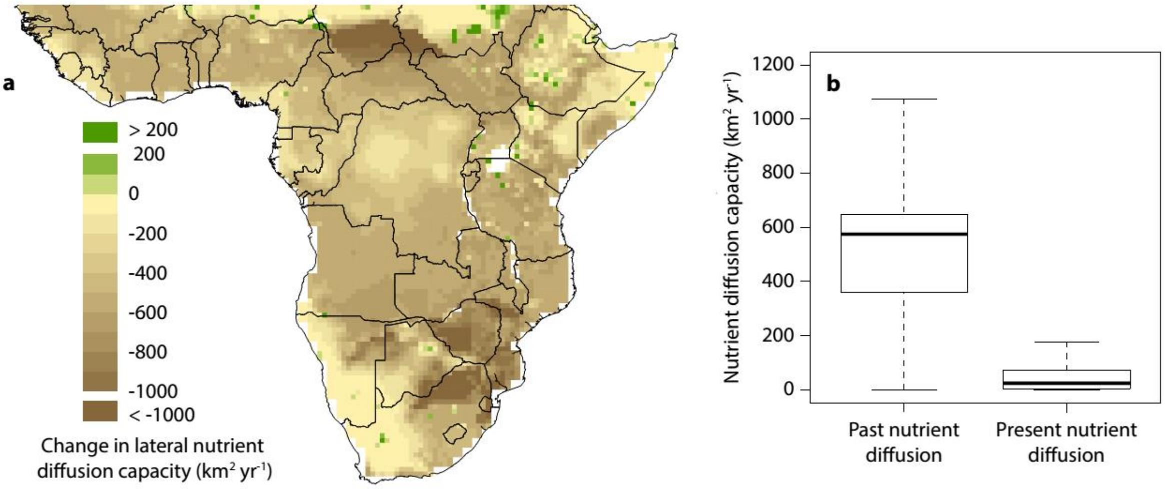

Figure S2. Lateral nutrient diffusion capacity without daily movement range modification. (**a**) Spatial variation in the change in nutrient diffusion capacity (km^2 yr^{-1}) at 0.5° grain, and (**b**) a comparison of past and present nutrient diffusion values. In (**b**), box shows the median and interquartile range of nutrient diffusion capacity for all sub-Saharan Africa 0.5° cells, and whiskers extend to the most extreme data point which is no more than 1.5 times the interquartile range. The map was generated using R version 3.3.3[41] (www.R-project.org) and QGIS 2.4.0[42] (www.qgis.org).
